# Impact of COVID-19 on Mothers Raising Children with Special Needs: Insights from a Survey Study

**DOI:** 10.3390/jcm12165363

**Published:** 2023-08-18

**Authors:** Lorenza Tiberio, Chiara Massullo, Giuseppe Carrus, Stefano Mastandrea, Sabrina Fagioli

**Affiliations:** Laboratory of Experimental Psychology, Department of Education, “Roma Tre” University, via del Castro Pretorio 20, 00185 Rome, Italy; lorenza.tiberio@uniroma3.it (L.T.); chiara.massullo2@uniroma3.it (C.M.); giuseppe.carrus@uniroma3.it (G.C.); stefano.mastandrea@uniroma3.it (S.M.)

**Keywords:** special needs children, COVID-19, neurodevelopmental disorders, mental health, distance teaching, lockdown, remote working

## Abstract

Home confinement during the COVID-19 outbreak had psychological effects that continue to be explored by researchers. This study investigated factors influencing the mental health of mothers caring for special needs children in Italy’s first lockdown. Specifically, we investigated the relationships between emotional states of depression, anxiety, stress, perceived distress related to home confinement, coping strategies, and other contextual variables (such as opportunities for distance learning and remote working) in a group of 68 mothers of children with special needs and 68 matched mothers of typically developing children. Data from an online survey showed no significant difference between the two groups. However, the research revealed that being a remote worker was a significant predictor of reduced stress in mothers of children with special needs, while distance learning was a significant predictor of reduced stress in mothers of typically developing children. In addition, the study found that hyperarousal symptoms were predictive of stress in mothers of children with special needs, while intrusive thoughts and avoidance coping were predictive of stress in mothers of typically developing children. In conclusion, further research is needed to develop effective support and intervention strategies for families with children with special needs and to deeply investigate the impact of flexible work arrangements and social aid on the mental health of mothers in non-emergency settings.

## 1. Introduction

It is still a common topic of conversation in our society to remember public health measures taken during the COVID-19 outbreak. Measures such as home confinement, social distancing, quarantine, and school closures were implemented to curb the spread of the virus, and while they were successful in doing so, research has shown that they also had a detrimental effect on individuals’ mental health. Brooks et al. [1] stated at the beginning of the outbreak that “successful use of quarantine as a public health measure requires us to reduce, as far as possible, the negative effects associated with it”. Several studies have documented that individuals forced to stay home during previous pandemics, like SARS and Ebola, frequently experienced multiple psychological symptoms such as anger, confusion, and post-traumatic stress [1,2]. A similar increase in psychopathology symptoms has been reported during the COVID-19 outbreak, including those related to depression, anxiety, addictions, and post-traumatic stress disorder (PTSD) (e.g., [3,4,5,6,7]), as well as a small increase in sales of psychotropic medications during the pandemic period (e.g., [8]). Conversely, certain psychological factors, like specific emotion regulation and coping strategies, appear to be linked to reduced negative psychological outcomes during the COVID-19 lockdown [9,10]. 

In the context of the COVID-19 pandemic, the physical and psychological health of children has been widely discussed. While children are typically less affected by the direct consequences of the virus compared to adults, they have still experienced indirect consequences on their psychological well-being. One of the first European studies examining the impact of COVID-19 quarantine on children’s and adolescents’ emotional and behavioral well-being compared the perceptions of 1143 parents of Italian and Spanish children aged 3 to 18 years old. The results indicated substantial changes in children’s emotional state and behavior, as perceived and described by the parents, including difficulty concentrating, boredom, irritability, restlessness, nervousness, feelings of loneliness, uneasiness, and worries [11]. 

The measures implemented to control the spread of COVID-19 have resulted in a significant shift in social life [9], with one of the most notable consequences being the closure of schools, which are vital centers of social interaction. As a result, many children and adolescents have missed out on essential social contacts that are crucial for their development. The prolonged implementation of these public health measures has had a particularly significant impact on the lives of children, especially those who were already living in socially disadvantaged circumstances [12]. 

In an effort to address this situation, distance learning was introduced, but its effectiveness was not uniform across all groups, particularly younger children and individuals with neurodevelopmental disorders [13,14,15]. This measure has brought about a notable shift in the dynamic between children and their caregivers, as it has added an educational component to the already existing parental responsibilities. More specifically, families have had to take on the responsibility of managing their children’s education and activities while at home. In general, the task of helping children, especially younger ones, with their schoolwork fell more heavily on mothers. As a result, mothers had to restructure their work and daily routines to accommodate the new demands of their children, often to a greater extent than fathers and not without consequences on their physical and mental well-being. For example, data from an Italian survey show that, during home confinement related to COVID-19, as compared to fathers, mothers showed high levels of parental stress [16]. According to this, Sun and colleagues also reported a strong relationship between high parental distress and high behavioral problems in children during the COVID-19 outbreak. 

Given the reasons and implications discussed so far, it is widely acknowledged that the period of home confinement has been a challenging and destabilizing time for most families. However, for families with children who have developmental disabilities and special educational needs, the effects of this situation have been especially pronounced [17]. Parenting a child with a neurodevelopmental disorder can be highly stressful, and the pandemic’s restrictive measures initially did not adequately address the needs of this already vulnerable population that requires additional support. For example, Neece, McIntyre, and Fenning [18] examined the impact of the first wave of COVID-19 by interviewing 77 families with young children with intellectual and developmental disabilities characterized by differences in ethnicity, language, and socioeconomic status. The results underline that the greatest challenge for these parents was to find themselves alone in the care and assistance of their children. As known, many care, educational, and social support services have been suspended during the lockdown period, and parents expressed concern about the long-term impacts of the pandemic on their children’s development. In England, Castro-Kemp and Mahmud [19] interviewed parents by means of an online survey. They found that a major part of parents of children with disabilities and/or special educational needs believed that school closures had a negative impact on their children’s mental health as well as on their own physical and mental health. Similar findings were found in Italy, where one of the earliest studies conducted during the first lockdown [20] involving 1126 parents suggested that parents of kids with psychological or physical disabilities reported significantly worsening behavioral issues in their kids, higher levels of parental burnout, and less perceived social support than parents of kids without disabilities. A cross-sectional study found that children with neurodevelopmental disorders experienced greater difficulties attending online classes and studying than controls, were less likely to miss their classmates, more frequently scolded by their parents, and caused more anxiety among their parents as compared to controls [21]. Several studies have examined how COVID-19, particularly the impact of pandemic restrictions, has affected the quality of life for school-age children and adolescents with autism spectrum disorder. Findings have consistently shown that these individuals have a higher likelihood of experiencing emotional, social, and behavioral difficulties than their neurotypical peers [22,23,24]. 

On the other hand, studies have demonstrated that different factors can predict the response to adverse events and mitigate the effects of social distancing consequences among vulnerable children. These factors include the child’s diagnosis, self-efficacy in parenting, and availability of educational support [25]. Additionally, research has suggested that caregiver depression, anxiety, and stress may contribute to an increase in the vulnerability of children with disabilities and their response to adversity when compared to children without disabilities [17].

Considering the existing research and the unique qualities of the enduring consequences of the COVID-19 pandemic, the aim of this study was to expand scientific knowledge pertaining to the impact of the COVID-19 outbreak on the experience of home confinement and the associated psychological implications among mothers of children with special needs, in comparison to mothers of children with typical development. First, we examined the psychological dimensions of mental health experienced by Italian mothers during the spring 2020 lockdown. Second, we investigated the predictors of mental health, including depression, anxiety, and stress, as they relate to coping strategies and responses to adversity. We also consider the influence of variables known to modulate the effects of home confinement, such as access to schooling facilities and agile working.

## 2. Materials and Methods

### 2.1. Participants

Data for the present study are part of a broad survey conducted in collaboration with UNICEF Italia (Available online: https://www.unicef.it/doc/9952/vita-famiglia-coronavirus-risultati-questionario.htm (accessed on 14 August 2023)). This national survey explored and investigated the psychological impact of the COVID-19 outbreak in Italian families with children of age between 18 months and 17 years. Data were collected during the last restrictive period of the lockdown established by the Italian Government. The initial sample was composed of 2473 parents. Of these, 2204 (82.1%) were mothers, 265 (10.7%) were fathers, and a small number (i.e., N = 4) were other reference figures (e.g., grandmother, aunt) that have lived with a minor during the lockdown. The average age of the total sample was 42 ± 6 years old (y.o.). Respectively, the average age was 41 ± 7 y.o. for the mothers and 45 ± 7 for the fathers. The vast majority of parents were of Italian nationality (97.2%), with a remaining percentage of 2.8% respondents of other nationalities (0.3%, respectively, Albania and Romania). For the current study, a sample of 68 mothers was selected based on their complete baseline data for the main study variables, ensuring that no data were missing.

These mothers (NEEDS+) have children with special needs, including intellectual disability (7.4%), autism (11.8%), specific learning disorders (SLD, 5.1%), movement disorders (7.4%), attention deficit hyperactivity disorder (ADHD, 3.7%), behavioral disorders (1.5%), genetic disorders (8.1%), or chronic illness (3.7%). NEEDS+ mothers were matched for age, educational attainment, occupational status, and child’s gender to 68 other mothers with typical developing children (NEEDS-). Mothers with typically developing children (NEEDS-) were selected from the same dataset using the Fuzzy command in the Python extension of IBM SPSS Statistics version 28. The selection process required absolute matching on educational attainment, occupational status, and child’s gender, with a matching criterion of a 10-year age range. 

### 2.2. Procedure

As face-to-face contact was not allowed, an online survey was implemented on the digital platform SurveyMonkey. The access link was shared through different methods: published on the main UNICEF Italy social networks, forwarded by email (to professors collaborating with the UNICEF “Scuole Amiche” Program, to the supporters of UNICEF Italy, and to the major associative and inter-associative realities focused on children and teenagers in Italy), and through WhatsApp. The survey link was active in the period between 21 April and 3 May 2020. Before completing the survey, information about the purpose of the study was provided, and participants then confirmed that they had understood the instructions correctly and agreed to participate. Data were collected anonymously (no name nor contact information were allowed) and treated for research purposes only. Volunteer participation in the study was acknowledged by both a positive answer to a direct question and submission of the questionnaire, which was not recorded if it was not completed.

### 2.3. Measures

In the survey, participants filled out questionnaires that assessed self-related psychological variables. These questionnaires were chosen due to the unique characteristics of the COVID-19 pandemic and, more specifically, the experience of being confined to one’s home.

#### 2.3.1. Impact of the Event Scale (IES) 

The immediate psychological impact of home confinement on the mothers included in the current study was assessed with the short version of the IES-6, which has been translated and adapted into Italian by Giorgi et al. [26]. The items of this scale were rated on a 4-point Likert scale and assessed the following aspects: (i) intrusion (i.e., intrusive thoughts, nightmares, intrusive feelings, and imagery), (ii) avoidance (i.e., avoidance of feelings, situations, and ideas), and (iii) hyperarousal (i.e., anger, irritability, hypervigilance, and difficulty concentrating). Total scores related to subjective stress range between 0 and 24, with high scores indicating a higher impact of the event in terms of stress. In our sample, the scale showed good consistency (Cronbach’s alpha: 0.846).

#### 2.3.2. Depression Anxiety Stress Scales (DASS)

Participants’ psychological distress was measured using the Italian version of the DASS 21 [27] Italian version of Bottesi et al. [28]. This scale assesses the negative emotional states of depression, anxiety, and stress present in the previous week. The scale foresees 21 items, which are answered on a 4-point Likert scale. Higher scores correspond to higher levels of distress, anxiety, and depressive symptoms. In our sample, the scale showed good consistency (Cronbach’s alpha: 0.942).

#### 2.3.3. Coping Orientation to Problems Experienced (COPE)

In addition, among the aims of the research, we explored how participants coped with stress related to the home confinement experience and the COVID-19 pandemic. Coping strategies were assessed using the COPE scale [29] in the Italian version (COPE-NVI; [30]). This scale includes 60 items. Rated on a 4-point Likert scale, each item asks participants to indicate how often they engage in a particular coping strategy when facing challenging or stressful situations. The items refer to five dimensions, composed of 15 subscales assessing a comprehensive range of coping strategies: social support (composed of comprehension and information seeking, and emotional expression); avoidance strategies (including denial, behavioral and mental disengagement, drug and alcohol abuse); positive attitude (composed of acceptance, positive re-interpretation, and restraint); problem solving (such as suppression of competing activities, planning, and activities); and turning to religion (composed of religion and absence of humor) [30]. In the current sample, Cronbach’s alpha was 0.612.

### 2.4. Statistical Analyses

The statistical analysis was conducted using IBM SPSS Statistics version 28. Descriptive statistics were used to examine the sociodemographic characteristics of the sample, and the chi-squared test was used to assess any differences between groups for the non-matched categorical variables. To assess differences in perceived emotional experiences between mothers of children with special needs and mothers of neurotypical children, we conducted paired *t*-tests on continuous variables, including IES, DASS, and COPE. 

In addition, we conducted Mixed Linear Models (MLM) to estimate the effects of other variables, such as distance teaching (DAD) and employment status (JOB), on the psychological dimensions used as indicators of mental health in this study. We examined whether the availability of distance online teaching contributed to overall distress across the groups. Indices of psychological distress (i.e., IES, DASS, and COPE) served as dependent variables in separate analyses within the statistical model. The group NEEDS+, NEEDS- was used as the grouping (independent) variable, and DAD was included as a covariate in the analysis. To account for the correlated observations within participants, a compound symmetry covariance matrix was employed, assuming consistent variance and covariance across the repeated measures. Model estimation was performed using Restricted Maximum Likelihood (REML), which adjusts for the fixed effects in the model and considers the degrees of freedom used for estimating those effects. We conducted a similar model to investigate the impact of employment status specifically focusing on being a remote worker, on the continuous dependent variables. This analysis was limited to mothers who worked from home (i.e., remote workers) and mothers who were not employed (e.g., homemakers, unemployed), had lost their job due to the lockdown, or were receiving income support due to temporary layoffs during the lockdown. By specifying the subject effect in the MIXED analysis, our statistical models accounted for the matched-pair design of the study, specifically the individual matching of NEEDS+ with NEEDS-. The level of statistical significance for all these analyses was defined as *p* < 0.05 (Bonferroni adjusted, see below).

As a second aim, we explored the predictors of mental health among mothers of children with special needs and mothers of neurotypical children, also considering the contribution of factors like access to schooling support and engagement in agile working as potential predictors of mental health. First, we evaluated the correlation between the response to an adverse event and emotional states of depression, anxiety, and stress and performed bivariate correlation analyses (Pearson *r*) separately for each group. Then, we examined the relationship between (1) avoidance, hyperarousal, and intrusion symptoms related to the COVID-19 emergency, as assessed by the IES scale; (2) coping strategies used to manage stress related to home confinement, as measured by the COPE scale; and (3) the level of emotional difficulties as measured by the DASS scale. Afterward, we conducted linear multiple regression analyses for each group separately, where we entered variables that were statistically significant after Bonferroni correction (*p* < 0.0019) as predictors. In each model, factors such as distance learning and employment status were included alongside the variables that were identified as significant in the previous correlation analysis. We used a probability of F-to-enter < 0.05 and a probability of F-to-remove > 0.10 for each multiple regression analysis. To incorporate the categorical variables as predictors in the regression model, “dummy variables” were created by assigning a new variable for each level of the categorical variable [31]. For example, for the predictor “Employment Status”, individual dummy variables were generated for each level of the variable, such as “remote workers” and “unemployed”. The variable takes the value of “1” for employed mothers in remote working and “0” for unemployed mothers. Similarly, for the predictor “Distance and At-home Dad” (DAD), a value of “0” was assigned to mothers who did not have distance teaching for their children, while a value of “1” was assigned to mothers who benefited from distance teaching for their children.

The level of statistical significance for all these analyses was defined as *p* < 0.05.

## 3. Results

### 3.1. General Characteristics of the Sample

The two groups of mothers were matched in terms of age, educational attainment, child’s gender, and employment status. The mean age of the sample was 41 ± 6 years, ranging from 25 to 56 years. Regarding educational attainment, 12% of mothers hold a basic school diploma, 44% have a secondary school diploma, 34% possess a bachelor’s or master’s degree, and 10% hold a post-university degree. At the time of the survey, 60% of mothers with special needs children were employed, encompassing full-time, part-time, and freelance positions. The remaining mothers were either stay-at-home moms (22%), unemployed (12%), or in other categories, such as receiving income support due to temporary layoffs. Sixty percent of mothers in both groups have a male child.

Ninety-one percent of the mothers in the control group and 82% of mothers with special needs children have a significant other, including those in marriages, cohabitation, or civil unions (χ^2^(5) = 5.929, *p* > 0.05). Approximately 28% of mothers in both groups have preschooler children (aged between 1.5 and 5 years), while the remaining 72% have school-aged children ranging in age from 6 to 18 years. 

In general, mothers in the two groups had differing perceptions of their professional and domestic workload before and during home confinement (χ^2^(2) = 7.371, *p* = 0.025). Specifically, mothers of children with special needs reported less often that their workload had decreased as compared to before the beginning of the lockdown (7% vs. 23% for mothers of typical developing children).

Among mothers with children who have special needs, 71% reported that the workload of caring for their child was significantly increased, 22% reported that it remained the same as before the lockdown, and only 4% reported that it was reduced to pre-confinement levels. Of these mothers, 68% reported not having any assistance, 12% had access to professional support, and 13% received help from family members living in the same household.

### 3.2. Comparing Perceived Distress, Emotional Difficulties, and Coping among a Sample of Mothers

#### 3.2.1. Impact of the Event Scale (IES) 

The pairwise *t*-test showed that there were no significant differences between the two groups on any of the dimensions of the IES scale (intrusion, avoidance, and hyperarousal). Means and standard deviations for the two groups on the IES scale are illustrated in Table 1.

Effect of Distance Teaching. The Mixed Model analysis, with the group NEEDS+ vs. NEEDS- as an independent variable and DAD as a covariate, did not yield any significant results on the IES scale after applying the Bonferroni correction.

Effect of Employment Status. A similar model that considered Employment Status as the covariate along with the independent factor Group did not yield any statistically significant results.

#### 3.2.2. Depression Anxiety Stress Scales (DASS)

Despite not observing statistically significant differences between the two groups on the DASS scale when applying the Bonferroni correction (*p* = 0.0125), it is noteworthy that there were significant differences observed in two specific dimensions: depression and the total scale. Specifically, when examining the depression dimension of the DASS, a significant difference emerged between the groups. Mothers with special needs children (NEEDS+) reported higher scores on the depression scale compared to the mothers with typically developing children (NEEDS-), indicating a higher level of depressive symptoms in mothers with special needs children. Additionally, a significant difference was found when considering the total scale score of the DASS, which encompasses depression, anxiety, and stress dimensions. The total scores were higher in the NEEDS+ group, indicating a greater overall psychological distress experienced by mothers with special needs children compared to the control group. Although these differences were statistically significant, it is important to interpret them cautiously, as the Bonferroni correction was not met. Nevertheless, the observed differences in depression and the total scale align with previous research findings [17], supporting the notion of increased psychological distress among mothers with special needs children during the lockdown.

It is noteworthy that both groups demonstrated scores that fall within the range indicative of mild depression (5–6) and mild anxiety (4–5). However, only mothers with children with special needs approached scores indicating moderate stress (10–12), surpassing the cut-off range for mild stress (8–9). The means and standard deviations for the two groups are illustrated in Table 1.

Effect of Distance Teaching. The analysis using Linear Mixed Models revealed a significant main effect of the covariate for the stress/tension dimension of the DASS scale (F = 12.473; *p* < 0.001). The covariate DAD was positively associated with DASS stress, indicating that the unavailability of distance online learning was related to higher levels of stress across the groups (Beta = 2.696; t(129.837) = 2.137; *p* < 0.05). Specifically, mothers whose children did not participate in online learning reported higher levels of stress (M = 11.37) compared to those whose children were engaged in distance education (M = 7.94). No other effects reached significance or survived Bonferroni correction in this analysis.

Effect of Employment Status. The analysis using the Linear Mixed Model considering Group as an independent factor and Employment Status as a covariate revealed a significant main effect of the covariate for the DASS anxiety (F = 8.036; *p* < 0.005), DASS depression (F = 6.997; *p* < 0.005), and DASS total (F = 6.986; *p* < 0.005). The analysis revealed that the covariate Employment Status was positively correlated with the outcome variable. Specifically, mothers who were not engaged in the form of agile work tended to exhibit higher levels of anxiety (Beta = 2.88; t(119.351) = 2.580; *p* < 0.05), depression (Beta = 2.693; t(112.32) = 2.312; *p* < 0.05), and overall distress (Beta = 8.636; t(112.032) = 2.769; *p* < 0.05) compared to mothers who worked from home. 

#### 3.2.3. Coping Orientation to Problems Experienced (COPE)

The results of the pairwise *t*-test indicated that there were no statistically significant differences between the two groups on any of the dimensions of the COPE scale. The means and standard deviations for the two groups are illustrated in Table 1.

Effect of Distance Teaching. The Mixed Linear Model analysis on the COPE scale, with Group as the independent variable and DAD as a covariate, did not reveal any significant results.

Effect of Employment Status. A similar model that considered Employment Status as the covariate along with the independent factor Group did not yield any statistically significant results after applying the Bonferroni correction.

### 3.3. Examining Predictors of Emotional Difficulties in a sample of Mothers

Crude univariate correlations between emotional difficulties and resilience factors are illustrated in Table 2.

Overall sample. All the DASS scores were positively correlated with the IES dimensions (intrusion, avoiding, hyperarousal, IES total score) and with avoidance strategies as measured by the COPE scale. No other significant correlations were found when considering all subjects together.

Mothers of children with special needs. The results of the correlation analysis found a positive association between scores on the DASS and IES scales for mothers of children with special needs. However, only scores related to intrusion, hyperarousal, and overall stress on the IES scale were found to survive the correction for multiple comparisons. Additionally, we found that symptoms of avoidance, as measured by the IES scale, positively correlated only with DASS scores for tension/stress and overall stress. The analysis also highlighted a positive association between DASS scores for depression and avoidance strategies, as measured by the COPE scale. No other correlations were found to be significant or survived the Bonferroni correction for multiple comparisons.

Mothers of children with typical development. The correlation analysis revealed a pattern of results comparable to the overall sample. 

#### 3.3.1. Predictors of Mental Health in Mothers of Children with Special Needs

Four linear multiple regression models were conducted to analyze the predictors of mental health in mothers of children with special needs. The models focused on the relationship between factors, such as the Impact of Event Scale (IES) and the COPE inventory, the availability of distance teaching and working from home, and their impact on DASS scores (depression, anxiety, stress, and total score). The results showed that the model for predicting depression symptomatology (DASS-Depression) had a significant regression equation (F(6,55) = 7.140, *p* < 0.001) with an R-squared value of 0.44. Regarding the individual contribution of each predictor, it is noteworthy that mothers with special needs children who worked from home reported a decreased DASS-Depression score of 1.97 compared to unemployed mothers (*p* = 0.055). Additionally, mothers who reported experiencing symptoms of physiological hyperarousal (IES-Hyperarousal; *p* = 0.057) and tended to use avoiding coping strategies (COPE-Avoiding; *p* = 0.059) had higher scores on the DASS-Depression scale.

The results of the multiple regression model for predicting anxious symptomatology (DASS-Anxiety) revealed a significant relationship, as indicated by a significant regression equation (F(5,56) = 7.588, *p* < 0.001) and an R-squared value of 0.40. The analysis found that Employment Status was a significant predictor of anxiety, with employed mothers reporting 2.25 points less on the DASS-Anxiety scale compared to unemployed mothers (*p* < 0.05).

Our regression analysis on symptoms of tension and stress revealed a statistically significant relationship between the variables under consideration (F(5,56) = 8.829; *p* < 0.001; R-squared value: 0.44). Further examination of the beta coefficients showed that employed mothers who worked from home had lower scores on the DASS-Stress scale compared to unemployed mothers (*p* < 0.05). Additionally, mothers who reported experiencing symptoms of physiological hyperarousal (IES-Hyperarousal; *p* < 0.01) had higher levels of stress.

Finally, we found a significant equation regression on the DASS total score (F(5,56) = 10.916; *p* < 0.001; R-squared value: 0.49). Again, we found that Employment Status and IES-Hyperarousal were significant predictors of overall emotional difficulties in mothers with special needs children, with *p*-values of less than 0.05 and 0.001, respectively.

The results of the linear multiple regression analyses are illustrated in Table 3.

#### 3.3.2. Typical Developing Mothers

Linear multiple regression models were used to analyze the factors that predict mental health in the control group, which consisted of mothers of typically developing children. The analysis revealed that the model for predicting depressive symptoms in these mothers was statistically significant (F(6,55) = 12.746; *p* < 0.001) and explained 58% of the variance in the data. One of the significant predictors of DASS-Depression was COPE-Avoiding (*p* < 0.001), with mothers who employed coping strategies based on avoiding reporting lower scores of DASS-Depression. 

The multiple regression analysis for predicting anxious symptoms (DASS-Anxiety) showed a significant relationship between the predictor variables and the outcome. This was evidenced by a significant regression equation (F(5,56) = 5.030, *p* < 0.001) and an R-squared value of 0.31, indicating that the model explains 31% of the variance in the data. However, the examination of individual predictors did not indicate any specific variable as a significant contributor to the outcome.

The model for predicting the symptomatology of tension/stress highlighted a significant regression equation (F(5,56) = 14.640; *p* < 0.001) with an R-squared value of 0.57. A closer examination of the beta coefficient showed that mothers who had access to distance teaching (DAD) for their children had 2.011 points lower scores on the tension/stress symptomatology scale compared to mothers who did not have access to remote instruction for their children (*p* < 0.05). Furthermore, mothers who reported experiencing intrusive thoughts related to the COVID-19 pandemic (IES-Intrusive) had higher levels of stress (*p* < 0.001).

In conclusion, the multiple regression analysis on the DASS total score (a measure of overall emotional difficulties) revealed a statistically significant relationship between the predictor variables and the outcome. The regression equation was significant (F(5,56) = 15.121; *p* < 0.001), and the R-squared value was 0.57, indicating that the model explains 57% of the variance in the data. One of the significant predictors of overall emotional difficulties in mothers of typically developing children was experiencing intrusive thoughts (IES-Intrusive; *p* < 0.001), indicating that mothers who reported experiencing such thoughts had higher scores on the DASS total scale.

The results of the linear multiple regression analyses are illustrated in Table 3.

## 4. Discussion

The purpose of this study was to examine the psychological consequences of home confinement due to the COVID-19 outbreak and their implications for mothers of children with special needs in comparison to mothers of typically developing children. Our results indicated no significant differences between the groups in terms of the perceived severity of the stressful situation or the strategies employed to cope with the stress. Interestingly, we found that mothers of children with special needs reported higher levels of psychological distress compared to mothers of typically developing children. While this outcome aligns with previous evidence, it is important to note that it did not meet the criteria for multiple comparison correction. Therefore, it should be interpreted with caution, considering the potential influence of other factors and the need for further research in this area. In addition, the findings of our survey revealed that accessing schooling support and engaging in agile working were associated with lower symptoms of psychological distress among mothers, regardless of whether they have a child with special needs to care for.

Remote work and distance learning have been the subject of significant empirical research exploring their impact on the well-being of families, particularly during periods of home confinement related to the COVID-19 pandemic [32,33]. As many countries have implemented lockdowns and physical distancing measures, remote work has become increasingly common. However, this shift has also been associated with challenges such as social isolation, increased workload, and stress for employees, requiring significant effort in terms of managing childcare, housework, and family responsibilities [34,35], especially for women [36]. On the other hand, a number of studies have highlighted the positive effects of remote working. These benefits include reduced stress related to commuting, increased flexibility, and improved work–life balance [37,38]. By allowing individuals to balance their work and personal life better, working from home has been shown to be helpful for reducing stress and improving mental health [39]. 

Research has demonstrated that distance education has yielded similar outcomes. Although it has been vital in ensuring educational continuity during the pandemic, there is conflicting evidence about its impact on the mental well-being of both children and their parents. Several studies have revealed that remote learning during COVID-19 has increased stress, anxiety, and depression among students [32,40], also due to the prolonged use of technology [11]. However, the impact of remote learning on mental health varies based on individual circumstances, as emotional difficulties may be exacerbated by pre-existing mental health problems, social isolation, or familial conflicts [41,42].

Interestingly, for many individuals who cannot typically attend in-person lessons, access to home schooling has represented a protective factor against poor mental health [43]. Additionally, socially isolated individuals have benefited from being socially connected with others and engaging in a community through remote learning [44].

Undoubtedly, the COVID-19 pandemic and related home confinement have presented numerous challenges for mothers, but having the opportunity to maintain their job and allowing their children to participate in distance learning appears to alleviate this burden in comparison to mothers without these opportunities, regardless of the effort required to care for children with special needs. As a result of providing flexibility and remote access to work and education, it is possible to assume that these approaches have preserved daily routines that are capable of reducing anxiety and uncertainty [32].

In addition to these findings, our analysis revealed specific predictors of mental health for mothers of children with special needs. We found that working from home was linked to lower levels of stress and anxiety compared to mothers who were unemployed. Additionally, mothers who reported symptoms of physiological hyperarousal related to confinement experienced higher levels of stress. Conversely, mothers of neurotypical children experienced reduced stress symptoms in relationship to the availability of schooling support for their children; however, they also experienced an increase in stress due to intrusive thoughts related to home confinement. Furthermore, we observed that mothers of neurotypical children who tended to use avoidance as a coping mechanism reported higher levels of stress. Overall, our findings suggested that various factors can be related to the mental health of mothers of children with special needs, and different coping strategies may have varying effects when compared to mothers of neurotypical peers.

Intrusive thoughts have been defined as unwelcome repetitive thoughts, images, or impulses [45] and have been associated with anxiety disorders [46]. They can be triggered by stress and can worsen hyperarousal symptoms, particularly when an effort is made to intentionally suppress them [47]. On the other hand, hyperarousal symptoms refer to a state of heightened physiological and emotional arousal. These symptoms manifest themselves as physical symptoms, such as a rapid heartbeat, sweating, or hyperventilation, as well as emotional symptoms, such as anxiety, fear, irritability, or anger. During stressful events, such as home confinement related to the pandemic, individuals use cognitive strategies to modulate their emotional responses, such as distraction or suppression, which involve diverting attention away from negative experiences to decrease emotional intensity [48]. However, the prolonged use of these strategies has been linked to increased physiological arousal. Studies suggested that cognitive reappraisal, which involves changing the interpretation of emotional stimuli or experiences, is a more effective and adaptive way to regulate emotions [49]. Research has shown that putting feelings into words can help regulate emotions by reducing activity in the amygdala, which is often hyperactive in individuals with anxiety and depression [50]. Therefore, maintaining social connections through technology and sharing emotions with significant others may be an effective way to reduce the intensity of negative experiences decreasing the intensity of hyperarousal symptoms. Individuals who have experienced significant stress or trauma in their lives may be more susceptible to both intrusive thoughts and hyperarousal, including mothers who care for children with special needs. This is especially true during the COVID-19 pandemic, where caring for children with special needs has been widely recognized as accompanied by significant distress for the caregiver [17,51]. In addition to the challenges of caregiving, mothers may also face additional challenges related to assisting with distance learning. With remote teaching, the child’s active involvement may be reduced, and parents may need to solve technical problems with the distance learning platforms, adding to the commitment of caregiving [40]. This may explain why these mothers did not benefit from distance learning in the same way as mothers of neurotypical children, for whom distance learning was a predictor of reduced stress. However, working remotely may have decreased stress for these mothers, as it provided an opportunity to divert attention from family duties.

In summary, caring for children with special needs can be a challenging and stressful task, especially during the COVID-19 pandemic. Mothers who care for children with special needs may be more susceptible to hyperarousal symptoms due to past stress related to caring for a child with difficulties. In addition, caregiving may be challenged by the need to assist with distance learning, which can reduce the child’s active involvement and add to the commitment to caregiving. While distance learning may not be as effective at reducing stress for these mothers as it is for mothers of neurotypical children, working remotely may provide an opportunity to divert attention from family duties and decrease stress levels. Mothers who care for children with special needs experienced substantial stress and difficulties, especially during the COVID-19 pandemic. Hence, healthcare professionals and policymakers must provide these caregivers with essential resources and support, such as social aid and flexible work arrangements. Additionally, it is important to ensure that children with special needs receive the necessary assistance to facilitate their learning, even in remote learning settings. By providing sufficient support to caregivers of children with special needs, the negative impacts of caregiving on their mental health and overall well-being can be reduced, which will ultimately benefit both the caregiver and the child.

This study has some limitations that should be taken into account. First, children with special needs encompass a wide range of conditions with varying degrees of severity. Due to this, the study’s findings may not be generalizable since it may be hard to disentangle specific effects of distance learning related to the specific condition. For example, children with chronic illnesses who cannot attend in-person school may have found advantages from distance learning, whereas children with autism spectrum disorder who need social contact could have suffered from remote education. These differential effects of distance learning on different conditions could have an impact on the mental health of mothers who are caring for these children providing, in turn, relief or burden of care. Another limitation of this study is that it did not consider the potential social support that the mothers may have had in their households. The study did not control for the number of children or other relatives living in the same house, which could have an impact on the mother’s caregiving burden and mental health. Future studies may benefit from collecting additional data on the social support available to mothers in their households to better understand the impact of distance learning and remote working on their mental health. Our study acknowledges the limitation of a small sample size, which may have limited the statistical power to detect smaller differences between the groups. In spite of this, it should be noted that the sample was obtained consecutively from a larger population of families with minor children. The selection of 68 families with a child with a disability was reflective of disability distribution among students in Italy. Data published by the Italian National Institute of Statistics indicate that 3.6% of enrolled students had a disability during the school year 20/21 [52]. Considering that our study selected 68 families with special needs from a sample of nearly 2500 families with minor children, representing approximately 3% of the whole sample, our sample can be considered representative of disabilities among Italian students.

A strength of this study is the homogeneity of the sample considered. While the consecutive nature of the sample did not allow for a homogeneous sample with respect to the specific condition of the children, the study was able to explore mental health issues related to caring for a child with special needs in a sample of mothers that matched a control group on several important variables. These variables include age; educational attainment; and some children’s characteristics, such as age and gender. This homogeneity of the sample can increase the internal validity of the study by reducing the impact of intervening variables, allowing for a more accurate assessment of the mental health of mothers in the two groups. In conclusion, our findings highlighted the potential impact of remote work and education on the mental health of mothers caring for children with special needs during the COVID-19 pandemic.

Further research in this area is warranted to fully understand the challenges faced by these families and to develop effective support and intervention strategies. This could involve investigating the impact of flexible work arrangements on the mental health of mothers caring for children with special needs, not only in emergency situations but also in non-emergency settings.

## Figures and Tables

**Table 1:** Mean and standard deviation of the Impact of Event Scale (IES), Depression Anxiety Stress Scale (DASS), and Coping Orientation to Problems Experienced (COPE) for mothers of special needs children and mothers of typically developing children.

	Special Needs Mothers (N = 68)	Typical Developing Mothers (N = 68)	t	*p*
*Impact of the Event Scale (IES)*	*Mean (S.D.)*	*Mean (S.D.)*		
IES Intrusion	3.97 (1.94)	3.60 (2.12)	1.114	0.269
IES Avoidance	3.40 (1.75)	3.24 (1.70)	0.555	0.581
IES Hyperarousal	3.65 (2.03)	3.18 (2.14)	1.332	0.187
IES Total Score	11.01 (4.87)	10.01 (5.09)	1.216	0.228
*Depression Anxiety Stress Scales (DASS)*				
DASS Depression	6.38 (5.12)	4.88 (4.53)	2.131	0.037
DASS Anxiety	5.22 (4.62)	3.87 (4.45)	1.819	0.073
DASS Tension/Stress	9.66 (5.49)	8.28 (4.68)	1.821	0.073
DASS Total Score	21.26 (13.99)	17.03 (11.95)	2.203	0.031
*Coping Orientation to Problems Experienced (COPE)*				
COPE Social Support	24.25 (6.31)	23.91 (6.51)	0.292	0.771
COPE Avoidance	24.28 (4.55)	23.88 (4.79)	0.550	0.584
COPE Positive Attitude	30.63 (6.46)	30.79 (5.88)	−0.160	0.873
COPE Problem Solving	26.21 (6.37)	25.46 (5.65)	0.673	0.503
COPE Turning to Religion	17.71 (4.07)	16.53 (3.07)	1.768	0.082

S.D. = Standard deviation.

**Table 2:** Bivariate correlation between resilience factors and emotional states of depression, anxiety, and stress for special needs and typical developing mothers.

	Special Needs Mothers (N = 68)				Typical Developing Mothers (N = 68)			
Impact of the Event Scale (IES)	DASS Depression	DASS Anxiety	DASS Stress	DASS Total Score	DASS Depression	DASS Anxiety	DASS Stress	DASS Total Score
IES Intrusion	0.467 * (<0.001)	**0.491 (<0.001)**	**0.473 (<0.001)**	**0.524 (<0.001)**	**0.611 (<0.001)**	**0.497 (<0.001)**	**0.694 (<0.001)**	**0.705 (<0.001)**
IES Avoidance	0.390 (0.002)	0.370 (0.003)	**0.468 (<0.001)**	**0.454 (<0.001)**	**0.439 (<0.001)**	**0.411 (<0.001)**	**0.589 (<0.001)**	**0.563 (<0.001)**
IES Hyperarousal	**0.567 (<0.001)**	**0.556 (<0.001)**	**0.559 (<0.001)**	**0.616 (<0.001)**	**0.480 (<0.001)**	**0.429 (<0.001)**	**0.485 (<0.001)**	**0.545 (<0.001)**
IES Total Score	**0.560 (<0.001)**	**0.558 (<0.001)**	**0.587 (<0.001)**	**0.626 (<0.001)**	**0.611 (<0.001)**	**0.531 (<0.001)**	**0.700 (<0.001)**	**0.720 (<0.001)**
Coping Orientation to Problems Experienced (COPE)								
COPE Social Support	−0.003 (0.983)	−0.078 (0.549)	0.045 (0.727)	−0.008 (0.952	0.100 (0.441)	0.212 (0.098)	0.308 (0.015)	0.243 (0.058)
COPE Avoidance	**0.411 (<0.001)**	0.224 (0.80)	0.269 (0.035)	0.332 (0.008)	**0.653 (<0.001)**	**0.627 (<0.001)**	**0.423 (<0.001)**	**0.666 (<0.001)**
COPE Positive Attitude	−0.202 (0.115)	−0.164 (0.203)	−0.107 (0.409)	−0.170 (0.186)	−0.073 (0.575)	−0.064 (0.624)	0.088 (0.495)	−0.019 (0.886)
COPE Problem Solving	−0.096 (0.457)	−0.084 (0.515)	−0.107 (0.409)	−0.106 (0.412)	−0.087 (0.500)	0.039 (0.761)	0.216 (0.092)	0.066 (0.611)
COPE Turning to Religion	0.101 (0.435)	0.132 (0.305)	0.151 (0.240)	0.142 (0.271)	0.014 (0.912)	0.063 (0.627)	0.002 (0.988)	0.031 (0.812)

* Pearson *r* (*p*-value). Significant correlations (Bonferroni corrected) are indicated in bold font.

**Table 3:** Multiple regression analysis and predictors of emotional difficulties in special needs and typical developing mothers.

	Special Needs Mothers (N = 68)				Typical Developing Mothers (N = 68)			
Independent Variables	DASS Depression	DASS Anxiety	DASS Stress	DASS Total Score	DASS Depression	DASS Anxiety	DASS Stress	DASS Total Score
	Beta (*p*) *	Beta (*p*)	Beta (*p*)	Beta (*p*)	Beta (*p*)	Beta (*p*)	Beta (*p*)	Beta (*p*)
Employment Status	−0.202 (0.055)	**−0.252 (0.020)**	**−0.206 (0.048)**	**−0.243 (0.015)**	−0.174 (0.054)	−0.150 (0.185)	0.001 (0.998)	−0.142 (0.111)
DAD	−0.092 (0.398)	−0.055 (0.615)	−0.208 (0.054)	−0.147 (0.148)	−0.070 (0.438)	−0.025 (0.827)	**−0.213 (0.021)**	−0.108 (0.229)
IES Intrusion	−0.044 (0.861)	0.121 (0.640)	−0.019 (0.901)	0.057 (0.693)	0.321 (0.177)	0.185 (0.535)	**0.468 (<0.001)**	**0.488 (<0.001)**
IES Avoidance	-	-	0.185 (0.160)	0.105 (0.401)	-	-	0.229 (0.057)	0.136 (0.249)
IES Hyperarousal	0.438 (0.057)	0.403 (0.083)	**0.426 (0.002)**	**0.481 (<0.001)**	0.120 (0.541)	0.074 (0.765)	0.078 (0.476)	0.180 (0.100)
IES Total Score	0.071 (0.849)	0.051 (0.893)	-	-	−0.016 (0.962)	0.289 (0.505)	-	-
COPE Avoidance	0.223 (0.059)	-	-	-	**0.430 (<0.001)**	-	-	-
R-squared	0.438	0.404	0.441	0.494	0.582	0.310	0.567	0.574
F	7.140	7.588	8.829	10.916	12.746	5.030	14.640	15.121
*p*	<0.001	<0.001	<0.001	<0.001	<0.001	<0.001	<0.001	<0.001
Constant	−2.315	1.394	5.847	0.459	−6.522	0.050	3.800	0.223

* *p* = 0.05. Significant predictors are indicated in bold font.

## Data Availability

The data presented in this study are available on request from the corresponding author.
